# CDC Grand Rounds: Promoting Well-Being and Independence in Older Adults

**DOI:** 10.15585/mmwr.mm6737a4

**Published:** 2018-09-21

**Authors:** Benjamin S. Olivari, Matthew Baumgart, Sarah L. Lock, C. Grace Whiting, Christopher A. Taylor, John Iskander, Phoebe Thorpe, Lisa C. McGuire

**Affiliations:** ^1^Emory University Rollins School of Public Health, Atlanta, Georgia; ^2^Division of Population Health, National Center for Chronic Disease Prevention and Health Promotion, CDC; ^3^Alzheimer’s Association, Chicago, Illinois; ^4^AARP, Washington, DC; ^5^National Alliance for Caregiving, Bethesda, Maryland; ^6^Office of the Associate Director for Science, Office of the Director, CDC.

Healthy aging is not merely the absence of disease or disability, but requires physical and mental health and ongoing social engagement (*1*). As the average U.S. life expectancy increases, recognition that public health can play a vital role in promoting healthy, successful aging even in the face of increased prevalence of chronic diseases, including types of dementia, among older adults (i.e., aged ≥65 years) has grown. Furthermore, actively engaging adults in prevention and wellness along with involving their caregivers (i.e., the family and friends of older adults who provide them with unpaid and informal support and services) can serve to prevent or delay the onset of physical disabilities and cognitive decline. Adults often are reluctant to discuss their concerns about worsening memory with their health care providers although such discussions can lead to earlier diagnosis and better care, planning, and support. As advances in public health and health care have helped increase life expectancy, public health professionals and health care providers have the opportunity to improve the quality of life for older adults and their caregivers and reduce the burdens associated with aging.

Each day, approximately 10,000 Americans reach age 65 years. By 2030, one in five Americans, 72.7 million, will be aged ≥65 years; this number is projected to reach 83.7 million by 2050. Within this group, the fastest growing age group will be persons aged ≥85 years, which is projected to increase from 5.9 million in 2012 to 8.9 million by 2030 (*2*). Longevity also provides advantages for society: Americans aged ≥50 years generate $7.6 trillion in economic activity each year (*3*). Along with benefits of longevity, however, the prevalence of chronic diseases (e.g., hypertension, diabetes, and arthritis) and other challenges increase with aging. Among adults aged ≥65 years, 80% have at least one chronic condition (*4*). Approximately one in three adults aged ≥65 years experiences limitations in their activities of daily living (e.g., eating, bathing, and dressing). One third of persons aged ≥65 years live alone, which can compound challenges associated with activities of daily living and increase social isolation risks (*5*,*6*).

To help address these challenges, in 2015, the National Prevention Council, chaired by the U.S. Surgeon General, developed the Healthy Aging in Action* report to identify recommendations and actions that promote healthy aging and improve health and well-being in later life (*6*). *Healthy Aging in Action* outlines strategies to eliminate health disparities, encourage safe and healthy communities, promote clinical and community preventive services, and empower older adults to make healthy decisions (*6*). One example of expanding older Americans’ access to clinical preventive services is through Vote & Vax.^†^ A community health organization known as Sickness Prevention Achieved through Regional Collaboration partners with many different collaborators at the federal, state, and local levels to increase the number of Americans who receive influenza vaccine by offering vaccination near polling places (*7*). In 2012, Vote & Vax served 651 polling locations across the majority of states and the District of Columbia. Approximately half (47.7%) of recipients reported that they had not received a flu shot the previous year or would not otherwise have been vaccinated. As well, 45% of persons receiving influenza vaccine at Vote & Vax clinics identified as African American or Hispanic, providing a potential strategy to reduce racial and ethnic disparities in receipt of influenza vaccination (*7*).^§^

Medicare, the primary health care payer for Americans aged ≥65 years, has incorporated prevention and screening services into two types of visits: the Welcome to Medicare visit and the Annual Wellness Visit. During the Welcome to Medicare visit, providers conduct a prevention-focused physical examination^¶^ and review beneficiaries’ medical and social history, risk for depression and mood disorders, functional ability, diet and physical activities, and their history of tobacco use (*8*). A written plan, similar to a checklist, is created to promote ongoing use of clinical preventive services and the discussion of important health topics, such as advance directives. Annual Wellness Visits** encompass personalized prevention plan services including a comprehensive health risk assessment, assessments to detect cognitive impairment, diabetes, hypertension, and missed vaccinations (*8*,*9*). However, these Medicare prevention and wellness benefits are not as widely used by older Americans as they could be; in 2013, only 6.8% of new Medicare enrollees took advantage of the Welcome to Medicare visit (*10*), and in 2014, approximately 16% of Medicare recipients had an Annual Wellness Visit; only an estimated 7% of Medicare beneficiaries receive all recommended preventive services (*9*,*11*). Annual Wellness Visit barriers include the relatively long duration of the visit (1 hour), low reimbursement rate for providers, and patient confusion about what is included in the visit (*11*). The U.S. Department of Health and Human Services established improving the rates of the Welcome to Medicare visits as an important *Healthy People 2020* objective (*10*).

## Healthy Body, Healthy Brain: The State of the Science and the Way Forward

Dementia is a general term used to describe symptoms characterized by the loss of cognitive function. Of the several forms of dementia, the most common is Alzheimer’s disease (*12*). Current estimates indicate that approximately 5.7 million Americans live with Alzheimer’s disease; it is the fifth leading cause of death for adults aged ≥65 years (*12*). African Americans and Hispanics have a higher risk for developing Alzheimer’s. From 1999 to 2014, the age-adjusted Alzheimer’s mortality rate increased 55%, from 16.5 to 25.4 per 100,000, and the number of deaths from Alzheimer’s increased 110%, from 44,536 to 93,541 (*13*). The high morbidity associated with dementia makes it the most costly disease in America (*14*). In 2017, caring for persons with dementia was estimated to cost the health and long-term care systems $259 billion. In addition, each year, approximately 15 million caregivers provide an estimated $230 billion in unpaid care (*14*).

Adults can, however, reduce their risk for, and lessen the impact and burden of, dementia. In 2015, both the Institute of Medicine and the Alzheimer’s Association independently concluded that regular physical exercise, smoking cessation, and the management of certain cardiovascular risk factors (e.g., diabetes, midlife hypertension, and midlife obesity) are steps that adults can take to lower their risk for cognitive decline (*15*,*16*). Another important step is to talk to a health care provider if worsening memory is a concern. Approximately half of the persons who are experiencing worsening memory have not talked about this concern with a health care provider (*17*). Early detection and diagnosis of dementia, as well as disclosure of the diagnosis to the patient and potential caregivers, are critical components of secondary prevention measures and facilitate accessing available treatments, building a care team, and improving medication management. Early diagnosis also can help persons with dementia and their families access support services, create advance directives, and address driving and safety issues (*18*).

Finally, care planning for adults with dementia can facilitate the coordination of care and improve its quality through better management of comorbid conditions. Better disease and medication management can result in fewer hospitalizations and emergency department visits (*19*). Medicare now covers care planning for persons with cognitive impairment. This includes evaluating cognition and function, assessing neuropsychiatric symptoms, evaluating safety, identifying the primary caregiver, and helping develop advance care directives (*20*).

## Healthy Caregiver, Healthy Patient: Importance of Healthy Aging for Caregivers

Informal or “family” caregivers are unpaid caregivers who provide care to a person, most often a relative, friend, or neighbor in the community or home setting, who needs assistance with activities of daily living and instrumental activities of daily living (e.g., preparing meals, shopping, or medical and nursing tasks). When backed by training and support (e.g., respite care), caregivers can help patients avoid unnecessary hospitalization and live in the community longer (*5*). Caregivers are not only critical in allowing adults to age within their chosen community; caregivers also can advocate on their behalf to health care providers and help manage medications, care plans, and transitions between care settings (*5*). On average, caregivers spend 24.4 hours each week providing help. Approximately one third of all caregivers are considered “high intensity caregivers” because they provide ≥21 hours of care weekly; on average, these caregivers provide 62.2 hours of care per week (*5*). Many caregivers, in addition to the hours they spend providing care, work in paid positions either full-time (34%) or part-time (25%) (*5*). Approximately one quarter (28%) of caregivers simultaneously provide care to an older adult in addition to raising their own children or grandchildren. These “sandwich generation” caregivers often also need to manage their own health, wellness, and financial needs because many are still in their prime working years before retirement (*5*). When providing care for persons with high-burden diseases (e.g., dementia and cancer), caregivers might experience declining health themselves. Many caregivers report high psychological stress and report an average of nearly $7,000 in out-of-pocket costs associated with caregiving each year (*5*,*21*).

Evidence-based interventions exist to promote the health and well-being of caregivers. The Resources for Enhancing Alzheimer’s Caregiver Health (REACH)^††^ program is an in-home, tailored, caregiver support intervention administered through the Department of Veterans Affairs, the Rosalynn Carter Institute, and other sites. REACH provides education and support for caregivers to improve overall caregiver health and reduce the burden from caregiving and the risk for depression (*22*). Identifying caregivers and assessing their stresses and needs can help maintain caregiver health and the health of the person receiving care and postpone costly alternatives such as placement in long-term care facilities (*23*).

Currently, only 15 states include family caregiver assessments within their Medicaid Home and Community-Based Services Waiver program, a program that supports persons who choose to receive long-term care services in their home or community rather than in an institutional setting (*23*). The Welcome to Medicare and Medicare Annual Wellness Visits also offer avenues for improving caregiver health and well-being by providing resources to caregivers who are supporting a patient with a chronic disease, including dementia (*8*).

## Public Health Activities and Programs

CDC’s Alzheimer’s Disease and Healthy Aging Program^§§^ developed important programs that focus on keeping older Americans healthy and independent (*24*). CDC’s Healthy Brain Initiative was established in 2005 through a Congressional appropriation (*24*). The Healthy Brain Initiative uses the tools of public health to catalyze action at state and local levels. *State and Local Public Health Partnerships to Address Dementia, The 2018–2023 Road Map,*^¶¶^ the third in the Road Map series, was released in 2018 and identifies 25 actions that state and local public health agencies and their partners can implement to promote cognitive health and address cognitive impairment and the needs of caregivers (*25*). The Road Map, which complements the National Plan to Address Alzheimer’s Disease, categorized the action items into four traditional domains of public health: monitoring and evaluation, education of the public, policies and partnership development, and assurance of workforce competency (*25*). The 2018–2023 Road Map action items emphasize diagnosis and disclosure of Alzheimer’s, risk reduction for Alzheimer’s, and caregiving for persons with Alzheimer’s.

In addition, CDC launched the Healthy Aging Data Portal,*** a free, publicly accessible online tool that provides data on essential indicators of health and well-being, including tobacco and alcohol use, screenings and vaccinations, mental and cognitive health, and caregiving at national, regional, and state levels (*24*). The Portal enables public health professionals and policymakers to examine a snapshot of the health of older adults in their states to prioritize and evaluate public health interventions.

Training and educating a workforce to work with older adults and those with Alzheimer’s disease is important (*4*). The Alzheimer’s Association, CDC, and Emory University’s Rollins School of Public Health Centers for Technical Assistance and Training has developed a curriculum^†††^ for undergraduate public health students that expands awareness of Alzheimer’s disease and related types of dementia as a growing, multilayered issue and is tied to the Core Competencies for Public Health Professionals (*26*). Health care providers are another important focus of education and training programs. The Gerontological Society of America developed the KAER toolkit^§§§^ to increase the use of evidence-based tools for assessing cognitive impairment by primary care providers and to promote better use of the Welcome to Medicare and Annual Wellness Visits (*27*). The toolkit improves detection of cognitive impairment and promotes earlier diagnostic evaluation and referrals for education and supportive community services for persons with dementia and their family caregivers.

Numerous opportunities exist to help promote the health, well-being, and independence of older Americans. Promoting available preventive services such as the use of Annual Wellness Visits and receiving all recommended vaccinations can improve well-being among older adults. Health care providers can use the tools discussed in this report to promote better health and care to ensure healthy aging for their patients. Federal, state, and local public health programs should employ approaches to optimize brain health and potentially prevent cognitive decline. Until better preventive strategies or therapies exist, public health professionals can disseminate and use data and tools from CDC’s Healthy Brain Initiative for the benefit of persons living with dementia and their caregivers.

## Footnotes

* https://www.cdc.gov/aging/pdf/healthy-aging-in-action508.pdf.

† http://www.voteandvax.org/.

§ The population of older adults is becoming increasingly more racially and ethnically diverse. From 2014 to 2060, the percentage of U.S. adults aged ≥65 who identify as non-Hispanic white is projected to decrease from 78.3% to 54.6%. https://assets.prb.org/pdf16/aging-us-population-bulletin.pdf.

¶ https://www.cms.gov/Outreach-and-Education/Medicare-Learning-Network-MLN/MLNProducts/downloads/MPS_QRI_IPPE001a.pdf.

** https://www.cms.gov/Outreach-and-Education/Medicare-Learning-Network-MLN/MLNProducts/downloads/AWV_chart_ICN905706.pdf.

†† http://www.rosalynncarter.org/rci_reach/.

§§ https://www.cdc.gov/aging.

¶¶ https://www.cdc.gov/aging/healthybrain/roadmap.htm/.

*** https://www.cdc.gov/aging/agingdata/index.html.

††† https://www.alz.org/media/Documents/curriculum-outline.pdf.

§§§ “Kickstart” the cognition conversation, “assess” for cognitive impairment, “evaluate” for dementia, “refer” for community resources (KAER). https://www.geron.org/programs-services/alliances-and-multi-stakeholder-collaborations/cognitive-impairment-detection-and-earlier-diagnosis.
